# Hollywood raising awareness of smoking-related diseases: can it proactively counteract the impact of smoking in movies?—the final mission of Star Trek’s Mr Spock

**DOI:** 10.1038/npjpcrm.2016.52

**Published:** 2016-10-20

**Authors:** Job FM van Boven, Alan G Kaplan

**Affiliations:** 1 Department of General Practice, Groningen Research Institute for Asthma and COPD (GRIAC), University Medical Center Groningen, University of Groningen, Groningen, The Netherlands; 2 Center for Pharmaceutical Outcomes Research, School of Pharmacy, University of Colorado, Denver, CO, USA; 3 Family Physician Airways Group of Canada, University of Toronto, Toronto, Ontario, Canada; 4 Department of Family and Community Medicine, University of Toronto, Toronto, Ontario, Canada

On 27 March 2015, generations of fans of *Star Trek* lost one of its iconic characters. That day, Leonard Nimoy, also known as Mr Spock on the starship *Enterprise*, died as a consequence of chronic obstructive pulmonary disease (COPD). What many people may not know about Leonard is that since his COPD diagnosis in 2013, despite giving up smoking about 30 years earlier, he became a *Star*-educator on his own disease. He reached out to his fans on Twitter and in interviews, discussing the burden of COPD, motivating people to quit smoking and highlighting his personal struggles while living with COPD. For a good reason: COPD is now the third leading cause of death in the world and is primarily caused by smoking exposure, but it is still one of the diseases that are least known by the general audience. After his death, his daughter and her husband continued his mission by announcing the film '*COPD: highly Illogical: a Special Tribute to Leonard Nimoy*', a documentary that will aim to educate viewers about COPD by using personal stories of Leonard, as well as information about treatments. It is expected to be launched in 2016.^1^ Ironically, many people may actually have initiated smoking because of television and Hollywood smoking influences.^2^ Therefore we were wondering whether we could also use movies and famous actors to educate their viewers on the health risks of smoking (i.e. the risk of lung cancer, cardiovascular disease and COPD) and actively involve Hollywood in helping them to quit—or never start—smoking?

The first question that arises: is there any scientific evidence that Hollywood can influence smoking behaviour? A PubMed search (performed on 28 March 2016) on smoking+movies resulted in 293 hits and the vast majority of relevant studies reported on the negative influence of depicting smoking in Hollywood movies. Several studies indicated that depiction of smoking in movies and TV dramas was associated with increased smoking rates in real life,^2–4^ recently confirmed by the US Surgeon General.^5^ By 2002, smoking exposure was even higher in youth-rated (G/PG/PG-13) movies than in adult movies (R).^3^ Policy regulations, such as the Master Settlement Agreement, that focused on restricting payments for tobacco placement in movies resulted in a lower proportion of movies and scenes with smoking.^6^ The latter is an interesting example of an externally initiated success intervention, with however a fairly passive role of Hollywood itself. Now the second question arises: Could we take it to the next level? That is, should we not only actively engage Hollywood actors and producers in avoiding exposure to smoking, but also stimulate them to proactively participate in awareness campaigns focusing on the health risks of smoking, in order to promote tobacco control? One could see it as a call upon the social responsibility of Hollywood to society.

Unfortunately, to the best of our knowledge, in scientific literature little is known about the educational and behavioural effects of raising awareness of the dangers of smoking and associated health risks by Hollywood. Short-term negative smoking consequences, such as coughing, were sometimes depicted, but few movies showed long-term negative health effects (e.g., lung cancer, COPD, cardiovascular disease).^3^ When chronic airway diseases got attention in movies, scenes mostly featured people suffering from asthma,^7^ a disease that is already widely known by the general audience but only partly related to smoking. Contrasting to the major worldwide health and economic impact of smoking-related diseases,^8,9^ a PubMed search (performed on 28 March 2016 by JFMvB) on the keywords COPD+movies yielded a mere nine hits, of which zero were considered relevant after reading the title and the abstract. Similarly, searches on lung cancer+movies or cardiovascular disease+movies returned few relevant hits.^10^ The searches were independently re-performed by the second author (AGK) (on 24 April 2016). No studies specifically reported on the effects of smoking-related disease education or awareness in mainstream TV series or movies on tobacco control.

Especially young adolescents, whose media saturation is most intense, are highly likely to be influenced by film characters as they often identify themselves with famous actors. Who did not want to be like—and act like—Johnny Depp, Britney Spears or Leonardo DiCaprio? Some of these stars smoke in films, but, for example, the notorious Pirate of the Caribbean also smokes in real life. Obviously, movie characters, and the actors and actresses who play them, are role models to many of us regarding the clothes we wear, what car we (want to) drive, the choice of our haircut, as well as our smoking behaviour. Actors’ and films’ potential role in disease education and healthy behaviours seems, however, underused.

There is plenty of evidence in the area of film and smoking initiation, but little evidence on the effects of depicting long-term smoking consequences on tobacco control. It may be plausible that we could use the smoking-in-movies evidence as an example strategy for how to promote, for example, COPD awareness. As a start, we would suggest that health organisations consider studying the historical relationship between Hollywood and the tobacco industry, as this clearly benefitted ‘big tobacco’.^11^ Their strategies included the hiring of professional product placement firms, placing their products in positive scenes, avoiding products in negative scenes and giving away free cigarettes to actors.^11^ Hopefully, a similar targeted strategy by health organisations may result in more awareness of the long-term smoking consequences and eventually better tobacco control. In this context, we could think of specific scenes in which long-term consequences of tobacco are depicted, but also meet-and-greets of patients with actors to increase their own awareness and make them tell their stories to their fans, just like Leonard Nimoy did.

You may all remember the episode ‘Trouble with Tribbles’. Would it not be great if we also remembered Mr Spock for his attempts to raise the odds for tobacco control? So far, he has made a first brave effort, but this hero unfortunately died in action. Who will be the next *Star*-educator following his courageous *Trek*?
