# Hollywood raising awareness of smoking-related diseases: can it proactively counteract the impact of smoking in movies?—the final mission of Star Trek’s Mr Spock

**DOI:** 10.1038/npjpcrm.2017.5

**Published:** 2017-06-22

**Authors:** Job FM van Boven, Alan G Kaplan

**Correction to**: *npj Primary Care Respiratory Medicine* (2016) **26**, 16052; doi:http://dx.doi.org/10.1038/npjpcrm.2016.52; published online 20 October 2016

This Editorial contained typographical errors in the main text.

‘On 27 March 2015, generations of fans of Star Trek lost one of its iconic characters’

Now reads:

‘On 27 February 2015, generations of fans of Star Trek lost one of its iconic characters.’

In the same section,

‘COPD: highly Illogical: a Special Tribute to Leonard Nimoy’

Now reads:

‘Remembering Leonard—His Life, Legacy and Battle with COPD’

These errors have now been corrected in the HTML and PDF versions of this Article.

